# Chikungunya Outbreak Risks after the 2014 Outbreak, Dominican Republic 

**DOI:** 10.3201/eid3012.240824

**Published:** 2024-12

**Authors:** Gideon Loevinsohn, Cecilia Then Paulino, Jessica Spring, Holly R. Hughes, Angela Cadavid Restrepo, Helen Mayfield, Michael de St. Aubin, Janeen Laven, Amanda Panella, William Duke, Marie Caroline Etienne, Gabriela Abdalla, Salome Garnier, Naomi Iihoshi, Beatriz Lopez, Lucia de la Cruz, Bernarda Henríquez, Margaret Baldwin, Farah Peña, Adam J. Kucharski, Marietta Vasquez, Emily Zielinski Gutiérrez, Aaron C. Brault, Ronald Skewes-Ramm, Colleen L. Lau, Eric J. Nilles

**Affiliations:** Massachusetts General Hospital, Boston, Massechusetts, USA (G. Loevinsohn); Brigham and Women’s Hospital, Boston (G. Loevinsohn, M. de St. Aubin, M.C. Etienne, G. Abdalla, S. Garnier, N. Iihoshi, M. Baldwin, E.J. Nilles); Ministry of Health and Social Assistance, Santo Domingo, Dominican Republic (C. Then Paulino, L. de la Cruz, B. Henríquez, F. Peña, R. Skewes-Ramm); Centers for Disease Control and Prevention, Fort Collins, Colorado, USA (J. Spring, H.R. Hughes, J. Laven, A. Panella, A.C. Brault); University of Queensland, Brisbane, Queensland, Australia (A. Cadavid Restrepo, H. Mayfield, C.L. Lau); Harvard Humanitarian Initiative, Cambridge, Massachusetts, USA (M. de St. Aubin, M. Baldwin. E.J. Nilles); Pedro Henríquez Ureña National University, Santo Domingo (W. Duke); Centers for Disease Control and Prevention, Central America Regional Office, Guatemala City, Guatemala (B. Lopez, E. Zielinski Gutiérrez); London School of Hygiene and Tropical Medicine, London, UK (A.J. Kucharski); Yale School of Medicine, New Haven, Connecticut, USA (M. Vasquez); Harvard Medical School, Boston (E.J. Nilles)

**Keywords:** chikungunya, CHIKV, viruses, vector-borne infections, arboviruses, outbreak, epidemiology, Caribbean, Dominican Republic

## Abstract

The 2014 chikungunya outbreak in the Dominican Republic resulted in intense local transmission, with high postoutbreak seroprevalence. The resulting population immunity will likely minimize risk for another large outbreak through 2035, but changes in population behavior or environmental conditions or emergence of different virus strains could lead to increased transmission.

In early 2023, a substantial increase in chikungunya disease cases in South America prompted an alert from the Pan American Health Organization (*1*). Although most chikungunya virus (CHIKV) transmission has occurred in Paraguay and Brazil, the proximity of nearby regions, including the Caribbean, with histories of intense arboviral transmission raises the prospect of epidemic spread. The Caribbean is an ecologically receptive setting for CHIKV and was heavily impacted by a 2014 epidemic (*2*). The epidemic was particularly severe in the Dominican Republic, where >539,000 cases and more deaths per capita than any other country in the Americas were reported (*3*,*4*). Given that vulnerability, we conducted a study to assess the risks for future CHIKV outbreaks in the Dominican Republic by evaluating post-2014 CHIKV transmission, estimating current population-level immune protection, and modeling future epidemic risks. Our research aims to inform public health interventions and preparedness strategies in anticipation of potential regional CHIKV resurgence.

Data and specimens were collected as part of a US Centers for Disease Control and Prevention–funded acute febrile illness (AFI) research program. The studies were approved by the Dominican Republic National Council of Bioethics in Health (#013-2019); the institutional review board of Pedro Henríquez Ureña National University (Santo Domingo, Dominican Republic), and the Massachusetts General Brigham Human Research Committee (Boston, MA, USA) (#2019P000094). 

## The Study

To understand local CHIKV epidemiology and transmission after the 2014 outbreak, we first performed reverse transcription PCR (RT-PCR) on acute-phase serum samples from patients enrolled as part of a prospective AFI surveillance program at Dr. Toribio Benosme Hospital in Espaillat Province in northwestern Dominican Republic and Dr. Antonio Musa Hospital in San Pedro de Macorís Province in southeastern Dominican Republic, using study processes described elsewhere (*5*). We invited patients ≥2 years of age with measured (≥38°C) or reported undifferentiated fever to participate. All adult participants and parents or legal guardians of child participants provided written consent; children 7–17 years of age provided assent. During November 2019–June 2023, we enrolled and tested 2,792 persons for a range of pathogens, including CHIKV, by RT-PCR (Appendix Table 1). We detected no cases of acute CHIKV infection, which aligns with national reported surveillance data that suggest minimal post-2014 transmission (Appendix Figure 1). 

**Figure 1:**
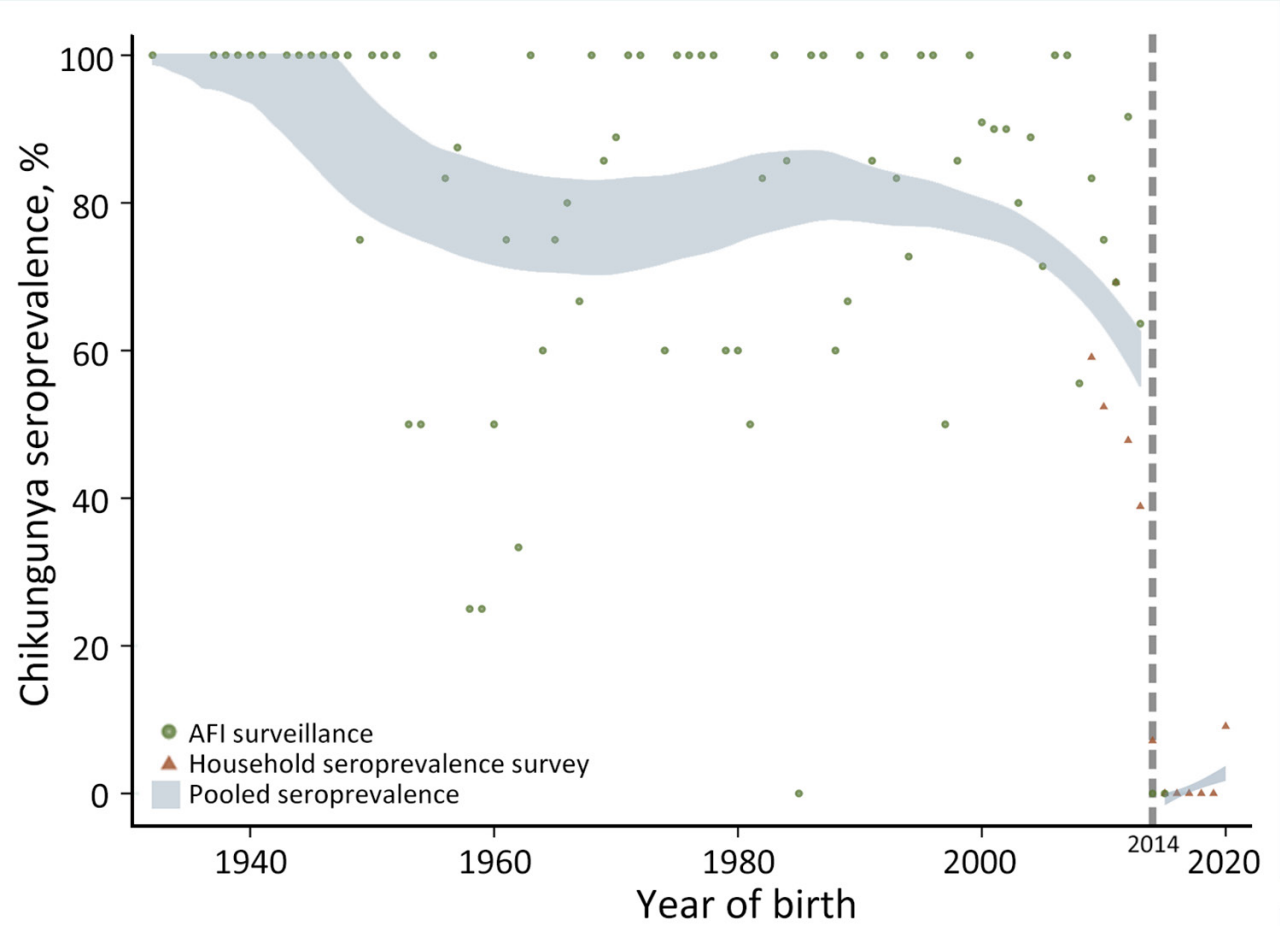
Chikungunya seroprevalence by year of birth in study of chikungunya outbreak risks after the 2014 outbreak, Dominican Republic. Blue band represents the 95% CI for pooled seroprevalence, which combined data from acute febrile illness and serosurvey cohorts. Estimates were obtained using kernel-weighted local polynomial smoothing weighted by the size of each birth cohort. AFI, acute febrile illness.

Next, to estimate population-level chikungunya seroprevalence, we conducted serologic screening among asymptomatic persons enrolled in a cross-sectional household cluster survey during July–October 2021 that included San Pedro de Macoris and Espaillat Provinces, which aligned with locations from the AFI surveillance program. The 3-stage cross-sectional sampling strategy, which enrolled household members ≥5 years of age, has been described elsewhere (*6*). We screened 201 serum samples using plaque reduction neutralization tests (PRNT) and 196 using ELISA IgG tests (Appendix). Of 397 persons enrolled from the 2 provinces, 319 (80.4%) were seropositive (Table). After adjusting for sex, age, and urban/rural setting, the estimated population seroprevalence across all age groups was 69.6% (95% CI 64.5%–74.8%) in 2021. That estimate based on national surveillance data assumed that persons born after 2014 were seronegative (Appendix Figure 1). To further assess that assumption, and because few participants enrolled in the serologic survey were born after the 2014 outbreak, we conducted serologic screening on serum samples from children enrolled through the prospective AFI surveillance platform (*5*). That testing enabled us to assess seropositivity derived from infection after the 2014 outbreak. Date of birth relative to the 2014 outbreak strongly predicted serostatus. Of 275 children screened using PRNT, 110 were born during 2009–2013 (60 [55%] seropositive), 14 in 2014 (1 [7%] seropositive), and 151 after 2014 (1 [0.7%] seropositive) (Table; Figure 1). 

**Table:** Population characteristics and prevalence information from study of future risks for chikungunya outbreaks in the Dominican Republic*

Category	Household serologic survey†					Acute febrile infection illness‡			
	All study participants, n = 397		CHIKV seropositive, n = 319	CHIKV seronegative, n = 78		All study participants, n = 275		CHIKV seropositive, n = 62	CHIKV seronegative, n = 213
Province									
San Pedro de Macorís	311		260 (84)	51 (16)		131		34 (26)	97 (74)
Espaillat	86		59 (69)	27 (31)		135		25 (19)	110 (81)
Other	0		0	0		9		3 (33)	6 (67)
Year of birth									
Before 2014	392		319 (81)	73 (19)		110		60 (55)	50 (45)
2014	3		0	3 (100)		14		1 (7)	13 (93)
After 2014	2		0	2 (100)		151		1 (1)	150 (99)
Median age, y (IQR)	32 (17.4–55.5)		31.9 (18–55.5)	32.7 (13–56.3)		6.7 (4.0–10.0)		10.7 (9.5–11.4)	5.3 (3.7–7.5)
Age group, y									
1–5	0		0	0		118		1 (1)	117 (99)
6–10	43		28 (65)	15 (35)		114		37 (32)	77 (68)
11–20	95		81 (85)	14 (15)		43		24 (56)	19 (44)
21–40	99		81 (82)	18 (18)		0		0	0
41–60	87		68 (78)	19 (22)		0		0	0
≥61	73		61 (84)	12 (16)		0		0	0
Sex									
F	250		206 (82)	44 (18)		131		37 (28)	94 (72)
M	147		113 (77)	34 (23)		144		25 (17)	119 (83)
Place of birth									
Dominican Republic	384		309 (80)	75 (20)		270		61 (23)	209 (77)
Other	13		10 (77)	3 (23)		5		1 (20)	4 (80)
Educational attainment									
No formal education	23		17 (74)	6 (26)		153		12 (8)	141 (92)
Primary	112		95 (85)	17 (15)		119		49 (41)	70 (59)
Secondary	123		104 (85)	19 (15)		2		1 (50)	1 (50)
Technical	13		11 (85)	2 (15)		0		0	0
University	37		26 (70)	11 (30)		0		0	0
Missing or not available	89		66 (74)	23 (26)		1		0	1 (100)
Occupation									
Active worker	91		67 (74)	24 (26)		1		1 (100)	0
Houseperson	82		72 (88)	10 (12)		0		0	0
Student	130		102 (78)	28 (22)		161		58 (36)	103 (64)
Retired	12		10 (83)	2 (17)		0		0	0
Unemployed	79		67 (85)	12 (15)		3		0	3 (100)
Preschool	0		0	0		110		3 (3)	107 (97)
Other	3		2 (67)	2 (67)		0		0	0
Self-reported prior infection									
Chikungunya	108		97 (90)	11 (10)		NA		NA	NA
Dengue	14		14 (100)	0		NA		NA	NA
Zika	3		3 (100)	0		NA		NA	NA

*Values are no. (%) except as inidicated. CHIKV, chikungunya virus; NA, not applicable. †Serologic status was assessed for household serologic survey participants using chikungunya plaque reduction neutralization test (n = 202) and ELISA to detect CHIKV IgG (n = 195). ‡Serologic status for all acute febrile illness surveillance participants was assessed using chikungunya plaque reduction neutralization test.

**Figure 2:**
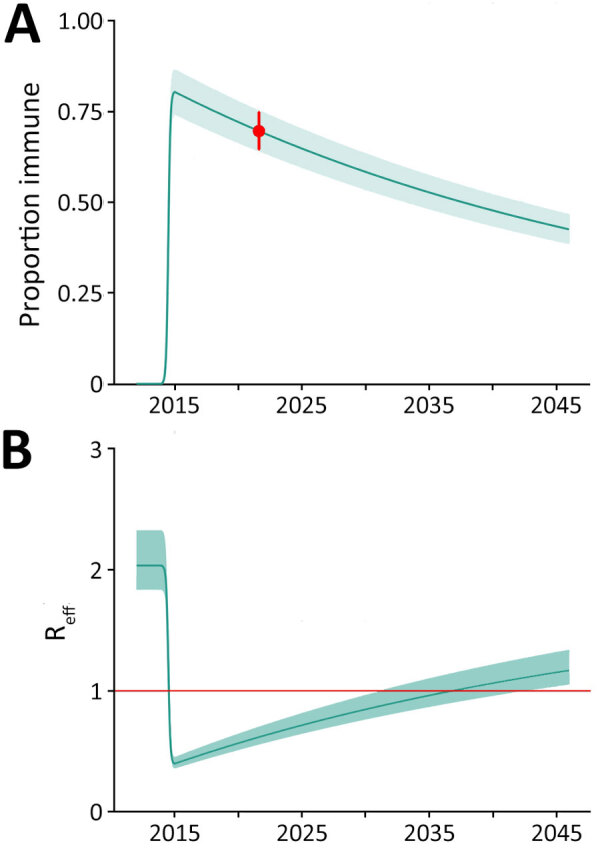
Projected chikungunya population immunity and R_eff_ in study of chikungunya outbreak risks after the 2014 outbreak, Dominican Republic. A) Estimated population immunity from 2012 through 2045 using a simulated population parameterized to the current population seroprevalence (red dot). Solid line represent point estimates and shading 95% CIs. Changes in population immunity over time reflect the introduction of new susceptible persons through births and decrease in immune persons through deaths. B) Projected changes in effective reproductive number over time calculated from the basic reproduction number R_0_ and population immunity. Solid line represents change in R_eff_ and shading 95% CIs, based on the simulated proportion of the immune population. The solid red horizontal line at R_eff_ = 1 represents the threshold for sustained transmission; values above this line indicate R_eff_ >1, suggesting potential for ongoing transmission, wheras values below this line indicate R_eff_ <1, suggesting a decline in transmission. R_eff_, effective reproduction number.

We then used our seroprevalence estimates to model population-level immune protection from 2012 through 2045 using methods reported elsewhere (*7*). We considered that infection generates lifelong immune protection and adjusted population immune protection over time to account for new births adding susceptible persons to the population and deaths reducing the pool of immune persons (Figure 2). Given minimal post-2014 transmission, we assumed no additional population immunity was generated after 2014. Based on our seroprevalence values, we calculated a basic reproduction number (R_0_) of 2.0 (95% CI 1.84–2.33). We calculated the effective reproduction number (R_eff_) over time using the R_0_ and population immune protection (Figure 2, panel A). Our findings suggest that the R_eff_ will remain <1.0 through 2035, indicating that, although clusters of CHIKV infection may occur, widespread and intense community transmission is unlikely. Finally, for comparison, we performed similar analyses for other Caribbean settings, including Jamaica and Puerto Rico, that have reported on CHIKV seroprevalence since the 2014 outbreak. Those analyses suggest that although postoutbreak seroprevalence and R_0_ values differ between settings (Appendix Table 2), the risk of widespread regional transmission will remain low through 2035 (Appendix Figure 2, panels A, B). 

## Conclusion 

We found serologic evidence of intense CHIKV transmission in study sites in northwestern and southeastern Dominican Republic during the 2014 outbreak but little subsequent transmission. Those findings were corroborated across a range of sources, including national surveillance, sentinel AFI surveillance, and a cross-sectional serologic survey. Our postoutbreak seroprevalence estimates provide key public health data for the Dominican Republic; the estimate (69.6%) was slightly lower than estimates from Jamaica (83.6%) and Haiti (78.7%) but higher than in other countries in the region (*8*–*11*). However, when differences in the timing of the serological surveys in relation to the 2014 outbreak are considered, and interval decreases in seroprevalence accounted for, post-2014 outbreak seroprevalence estimates across the 3 countries were broadly similar, and all were >80%. 

We concluded that the 2014 outbreak generated high levels of population immune protection. However, in the absence of meaningful ongoing transmission, population protection will decrease. Because new births add susceptible persons to the population and deaths reduce the pool of immune persons, the ratio of susceptible to immune persons increases over time. As this ratio increases, so does the risk for a sustained outbreak. Awareness of when population immune protection falls below a threshold that could allow widespread transmission is critical for public health forecasting and response, both in the Dominican Republic and in settings with similar immunologic and epidemiologic profiles. 

Our analyses suggest that population immune protection derived from the 2014 outbreak is likely to minimize the risk for another large outbreak through at least 2035 (Figure 2, panel A) if other factors remain unchanged. However, changes in population behavior or environmental conditions, or emergence of new strains, could lead to increased transmission, as documented in the 2022–2023 outbreak in Paraguay, when transmission expanded to multiple previously unaffected regions (*12*). 

Limitations included lack of generalizability of the data sources. We largely addressed this limitation by using multiple data sources to confirm key findings, with the exception of population seroprevalence estimates, for which we relied on a single source. The number of study participants was limited, and data were restricted to 2 provinces; therefore, our point estimates might not be representative of national seroprevalence. In addition, the specificity of CHIKV immunoassays may be affected by other circulating alphaviruses (*13*), although substantially lower seroprevalence among those born after 2014 suggests that effect was unlikely. 

In conclusion, chikungunya immune protection generated during the 2014 outbreak in the Dominican Republic will likely minimize the risk for widespread and intense transmission in the next decade, similar to findings from Jamaica and Puerto Rico. However, changes in behaviors, environmental conditions, or emergence of new strains could affect those predictions. Therefore, public health authorities should closely monitor chikungunya activity and develop preparedness plans to mitigate effects of future outbreaks. 

Appendix Additional details from study to assess the future risks for chikungunya outbreaks in the Dominican Republic
