# Supplementary material for: Chikungunya Outbreak Risks after the 2014 Outbreak, Dominican Republic
Source: Emerg Infect Dis. 2024 Dec;30(12):2679–83. doi: 10.3201/eid3012.240824 (PMC11616656; doi:10.3201/eid3012.240824)
Supplement: Appendix — Additional details from study to assess the future risks for chikungunya outbreaks in the Dominican Republic [file 24-0824-Techapp-s1.pdf]

Article DOI: https://doi.org/10.3201/eid3012.240824

EID cannot ensure accessibility for supplementary materials supplied by authors. Readers who have difficulty accessing supplementary content should contact the authors for assistance.

Chikungunya Outbreak Risks after the 2014 Outbreak, Dominican Republic

Appendix

Methods

Molecular detection of CHIKV

RNA was extracted from sera using the QIAamp Viral RNA Mini kit (QIAGEN). RNA extracts were then tested using real-time RT-PCR technique and a ZCD multiplex Zika, chikungunya, and dengue virus (DENV1-4; ZCD) assay on an ABI 7500 Fast (Applied Biosystems) using commercially available primers and probes. Real-time RT-PCR was performed according to the manufacturer's instructions.

IgG Enzyme-Linked Immunosorbent Assay (ELISA)

The IgG ELISA was performed as previously described and has been demonstrated to correlate closely with plaque-reduction neutralization assays (1). Briefly, the inner 60 wells of a 96 well flat-bottom plate (Immulon II HB, ThermoFisher) were coated with the alphavirus cross-reactive monoclonal antibody 1A4B-6 (Arboviral Diseases Branch, Arboviral Reference Collection) diluted in carbonate coating buffer and incubated overnight at 4°C. The coating buffer was removed, and plates blocked with blocking buffer (PBS, 0.1% Tween20, 3% goat serum [Colorado Serum Co.]) for 1 hour at room temperature. Plates were washed 5 timers with PBS, 0.05% Tween20 wash buffer. CHIKV suckling mouse brain antigen (Arboviral Diseases Branch, Arbovirus Reference Collection) was diluted in wash buffer and 50 µl added to 3 wells per test sample, normal mouse brain antigen was also added to 3 wells per test sample, and incubated overnight at 4°C. Antigens were washed 5 times with wash buffer, and test serum was diluted 1:400 in wash buffer, 50 µl added to 6 wells (3 wells of CHIKV antigen and 3 wells of negative antigen), and incubated for 1 hour at 37C in a humid chamber. Known positive control

serum and normal human serum were included as controls. The plates were washed 5 times, and 50 µl alkaline-phosphate conjugated goat anti-human IgG (Jackson Immunoresearch) was added to the wells and incubated for 1 hour at 37°C in a humid chamber. The plates were washed 10 times and developed with 3 mg/ml p-nitrophenyl phosphate disodium (Sigma) in 1M Tris base pH 8.0. for 30 minutes at room temperature. 3M sodium hydroxide was added to stop the development and plates were read in a plate reader (BioTek) at 405nm. Positive samples were determined by the mean OD of the sample serum on viral antigen/the mean OD of the negative human serum on viral antigen (P/N). A P/N greater than 2.0 is considered positive.

Plaque reduction neutralization test ($PRNT_{90}$)

Serum samples were screened for neutralizing antibodies against CHIKV East, Central and South African- Indian Ocean (ECSA-IOL) strain 91064 (GenBank accession EF451144) in Vero cells (ATCC CCL-81). Briefly, samples were diluted 1:10 in BA-1 (M199-H, Sigma-Aldrich; 0.05 M Tris-HCl, Invitrogen; 1% bovine serum albumin, Millipore) and heat inactivated at 56°C for 30 minutes. 60 µl of diluted serum was incubated with an equal volume of CHIKV at 200 plaque forming units (pfu)/0.1 mL supplemented with 8% Labile Serum Factor (LSF) (Millipore) for one hour at 37°C. Back titrations of 10-fold dilutions, representing 100 plaques, 10 plaques, and 1 plaque were also incubated for one hour at 37°C. 100 µl of sample/virus and back titration were added to Vero cells seeded in 6-well cell culture plates. Plates were incubated for one hour at 37°C then overlayed with 3 mL of yeast extract-lactalbumin (Ye-Lah) media containing 0.5% (w/v) Agarose (Lonza), 3% (v/v) sodium bicarbonate (Invitrogen), 2% (v/v) FBS (Hyclone), 0.1% (v/v) Gentamicin (MP Biomedicals), 0.4% (v/v) Fungizone (Hyclone), and 1% (v/v) Pen/Strep (Invitrogen). Two days post incubation, a second Ye-Lah overlay with 3% (v/v) neutral red (Sigma-Aldrich) was applied. Plaques were counted the next day. Samples were compared to the back titration and considered positive if they had ≥90% neutralizing at 1:20.

Calculation of population immunity

We conducted a time-series analysis from January 1, 2012, to December 31, 2045, to examine the dynamics of immunity and the effective reproductive number in a population (Fig 1). An initial population of 1,000 persons were considered, with varying birth and death rates from 2012 through 2045. We assumed interval birth and death rates were linear between these timepoints. We also assumed that prior to 2014 the population was fully susceptible. A sigmoid function was applied in 2014 to simulate a rapid increase in immunity. Post-2014, adjustments

were made to the immune population by adding new births to the susceptible pool and removing persons that died from the immune pool. Data source for country-specific births and deaths was the United Nations World Population Prospects, accessed from http://www.macrotrends.net/countries.

Calculation of R_0 and R_{eff}

We estimated the basic reproduction number for different infection scenarios based upon the estimated seroprevalence of the simulated population following the outbreak, where χ = the final susceptible proportion of the population post-outbreak.

$$R_0 = -ln(\chi)/(1-\chi)$$

R_0 values were used to estimate the R_{eff}, which was calculated iteratively for each day of the dataset from January 1, 2012 to Dec 31, 2045, as follows, where *A* = number of immune persons and *B* = total simulated population.

$$\text{Reff} = R_0 \times \left(1 - \frac{A}{B}\right)$$

Times series of national reported cases

Using data on reported CHIKV cases provided by the Dominican Republic Ministry of Public Health and Social Welfare, we aggregated the case counts by week and year. Reported cases included both laboratory confirmed and suspected cases.

Data sources

National chikungunya surveillance data that includes reported cases from 2013 through 2021 were provided by the Dominican Republic Ministry of Public Health and Social Welfare. Data on birth and death rates are from the United National Department of Economic and Social Affairs, Population Division, and are available from https://www.macrotrends.net/countries/DOM/dominican-republic. Data on seroprevalence for Jamaica and Puerto Rico were obtained from the cited studies. All other data were enumerated through the current study.

Programming language

Analyses and data visualization were performed using the R statistical programming language (R version 4.2.3, 2023-03-01), with ggplot2 for data visualization.

Appendix Table 1. Acute Febrile Infection Surveillance Study Population (N = 2792)

Chraracteristic	Study participants, n (%)
Home province	
San Pedro de Macorís	1136 (41)
Espaillat	1487 (53)
Other	169 (6)
Median age, y (IQR)	27 (12–41)
Age range, y	
1–5	408 (15)
6–10	206 (7)
11–20	456 (16)
21–40	1006 (36)
41–60	534 (19)
≥61	182 (7)
Gender	
F	1615 (58)
M	1169 (42)
Other	1 (0)
Country of birth	
Dominican Republic	2701 (97)
Other	88 (3)
Unknown	2 (0)
Symptoms	
Chills	2202 (79)
Malaise	224 (8)
Headache	200 (7)
Cough	2121 (76)
Vomiting	62 (2)
Diarrhea	63 (2)
Myalgia	1835 (66)
Arthralgia	69 (2)
Erythematous rash	94 (3)

Appendix Table 2. Estimated CHIKV seroprevalence and basic reproductive numbers in the Dominican Republic, Jamaica, and Puerto Rico

Category	Dominican Republic	Jamaica	Puerto Rico
Year of survey	2021	2017–2019	2018–2019
Sample size	397	584	4,035
Estimated seroprevalence at the time of survey, % (95% CI)	69.6 (64.5–74.8).	83.6% (80.0–86.5)	31.4 (30.0–32.9)
Estimated seroprevalence at the end of 2014, % (95% CI)	80.6 (74.6–86.7)	88.6 (85.0–92.1)	33.6 (32.0–35.1)
Basic reproductive number (95% CI)	2.0 (1.84–2.33)	2.45 (2.23–2.71)	1.22 (1.21–1.23)

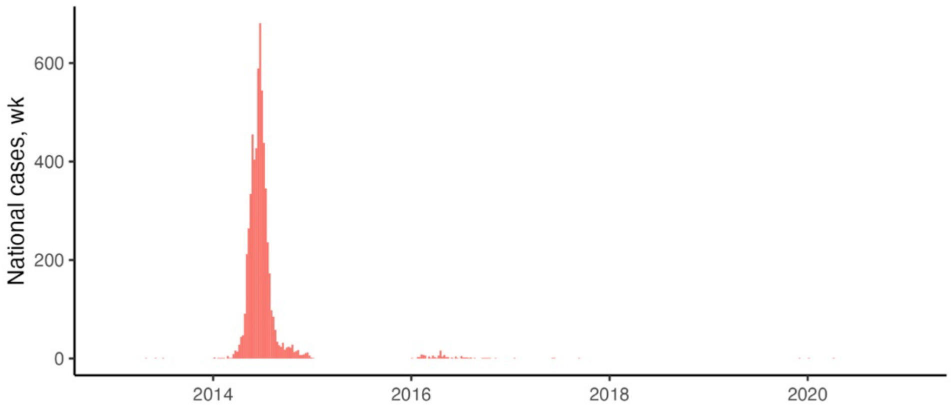

Appendix Figure 1. National reported CHIKV cases by week, Dominican Republic. X-axis tick marks indicate January 1 of the respective year. Data obtained from the Dominican Republic Ministry of Public Health and Social Welfare.

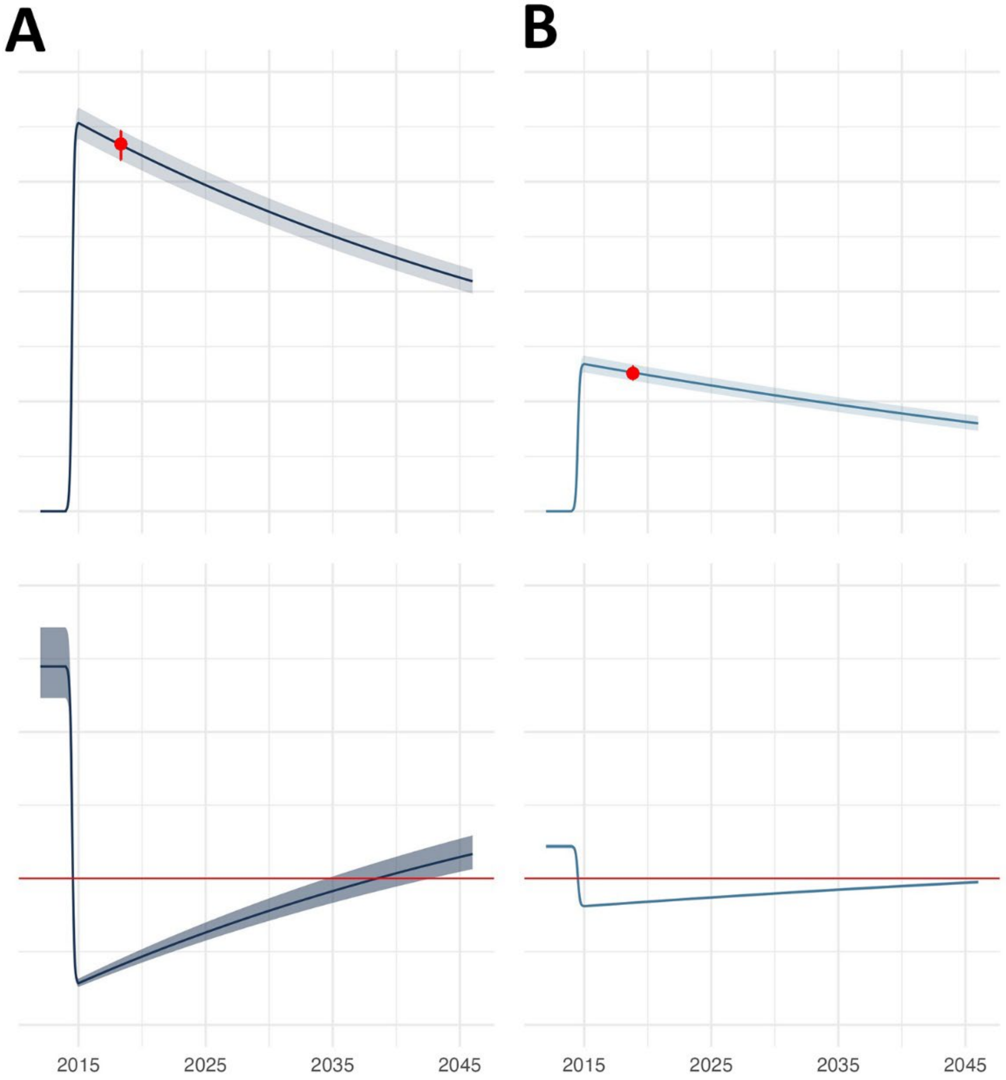

Appendix Figure 2. Projected chikungunya population immunity and effective reproduction number (R_{eff}) in other Carribean settings. Top row represents estimated population immunity during 2012–2045 using a simulated population parameterized to the current population seroprevalence (red dots) with 95% CIs (grey bands), with estimates from previously reported seroprevalence studies from A) Jamaica (among women attending antenatal care) and B) Puerto Rico (among a cohort of persons ≤50 years of age). Dark lines represents the point estimates with grey bands representing 95% CIs. Changes in population immunity over time reflect the introduction of additional susceptible persons through births and decrease in immune persons through deaths. Bottom row presents projected changes in R_{eff} over time calculated from R_0 and population immunity. Dark lines represent changes in R_{eff} based on the simulated proportion of the immune population shown in the top row.
